# Intimate partner violence and associated factors among pregnant women attending antenatal care service in Debre Markos town health facilities, Northwest Ethiopia

**DOI:** 10.1371/journal.pone.0218722

**Published:** 2019-07-01

**Authors:** Zelalem Nigussie Azene, Hedija Yenus Yeshita, Fantahun Ayenew Mekonnen

**Affiliations:** 1 Reproductive Health, School of Midwifery, College of Medicine and Health Sciences, University of Gondar, Gondar, Ethiopia; 2 Department of Reproductive Health, Institute of Public Health, College of Medicine and Health Sciences, University of Gondar, Gondar, Ethiopia; 3 Department of Epidemiology and Biostatistics, Institute of Public Health, College of Medicine and Health Sciences, University of Gondar, Gondar, Ethiopia; Liverpool School of Tropical Medicine, UNITED KINGDOM

## Abstract

**Background:**

Intimate partner violence is a thoughtful public health concern and human rights violation towards pregnant women for it has a significant negative health effect on the life of both the mother and her fetus. However, there is a scanty of information about the extent of intimate partner violence during pregnancy in Ethiopia, particularly in the study area. Therefore, the current study was conducted to determine the prevalence of intimate partner violence among pregnant women attending antenatal care and identify associated factors that cause it.

**Methods:**

An institution based cross-sectional study was conducted on 409 pregnant women who were attending antenatal care service in Debre Markos town from March 17, 2018 –April 28, 2018. Systematic random sampling technique was used to select study participants. A pre-tested structured questionnaire was used to collect the data. Bivariable and Multivariable logistic regression models were done. Adjusted odds ratio with 95% confidence interval was used to identify factors associated with intimate partner violence during pregnancy.

**Results:**

The prevalence of intimate partner violence during current pregnancy was found to be 41.1% (95% confidence interval (CI): 36.0–46.0). Of this, the prevalence of psychological, physical, and sexual violence was 29.1%, 21%, 19.8% respectively. Lower educational status of partners (AOR = 3.26, 95%CI: 1.45–7.36), rural residency (AOR = 4.04, 95%CI: 1.17–13.93), frequent alcohol abuse by partner (AOR = 4.79, 95% CI: 2.08–11.04), early initiation of antenatal care (AOR = 0.44, 95% CI: 0.24–0.81), the age of women between 17–26 years (Adjusted odds ratio (AOR) = 0.21, 95%CI: 0.09–0.49),choice of partner by the women only (AOR = 3.26,95% CI:1.24–8.57) were statistically significant factors associated with intimate partner violence towards pregnant women.

**Conclusions:**

In this study, the prevalence of intimate partner violence during pregnancy is found to be high. As a result, interventions that would address the above mentioned factors need to be implemented.

## Background

Intimate partner violence (IPV) can be manifested by acts of physical aggression, sexual coercion, psychological/emotional abuse or controlling behaviors by a current or former partner or spouse [1].

Intimate partner violence is a thoughtful public health concern and human rights violation towards pregnant women because of its huge negative health effect on the life of both the mother and her offspring [2].

Violence against women is largely recognized as a major human right abuse, and a significant public health problem with multiple adverse physical, mental, sexual, and reproductive health effects [3, 4]. Since pregnancy is a period that might demand increased relationship commitment and increase the resources needed, some risk factors are likely to be more important during pregnancy, thereby, causing the violence or aggravating it [5]. A number of mechanisms for how IPV may have an influence on low birth weight, small for gestational age and preterm births have been documented and include direct health, mental health, and behavioural effects, all of these help providers a lot to identify women who are at high risk of IPV during pregnancy [6]. Every year more than 324,000 women are estimated to have experienced IPV during pregnancy[7]. According to WHO multicounty study, the global prevalence of intimate partner violence (IPV) during pregnancy ranges from 1 to 28% [4] and it ranges from 2% to 57% in Africa [8]. Furthermore, the overall prevalence of intimate partner violence during pregnancy in developing countries is higher (27.7%) than that in developed countries (13.3%) [9].In Ethiopia, a community- based study revealed that 44.5% of women experienced intimate partner violence during pregnancy [10].

Intimate partner violence during pregnancy has been found to be associated with fatal and non- fatal adverse health outcomes of pregnant women and their offspring. These adverse health outcomes may be caused by direct injuries of physical abuse to a gravida as well as physiological effects of stress from present or previous abuse on fetal growth and development. Homicide and suicide which are fatal outcomes associated with IPV during pregnancy are the two most extreme consequences [11]. Non-fatal outcomes associated with IPV during pregnancy include adverse pregnancy complications (e.g., low birth weight, premature delivery, miscarriage, abortion, antepartum hemorrhage, intrauterine growth retardation and perinatal death), negative health behaviors (e.g., drug and alcohol abuse, smoking) and adverse psychosomatic outcomes (e.g., physical injuries, depression, anxiety and suicidal tendencies [11–19]. Previous studies identified that intimate partner violence against pregnant women significantly associated with alcohol drinking by a partner, educational status, residence, being in a polygamous union, multiparous, occupational status, violence during childhood, age of the women, dowry/bride payment [20–22].

In Ethiopia, even though studies were conducted on women, there is a dearth of information regarding intimate partner violence during pregnancy. Therefore, this study was aimed to assess the prevalence of intimate partner violence and associated factors among pregnant women.

## Methods

### Study design and period

Institutional based cross-sectional study was conducted from March 17, 2018 –April 28, 2018 in Debre Markos town. The town is located in East Gojjam zone in Amhara regional state, Northwest Ethiopia. It is 299kms far from Addis Ababa, the capital city of Ethiopia and 265km from Bahir Dar, the capital city of Amhara Regional state. According to the Population projection of Ethiopia for all regions at woreda level from 2014–2017, the total population of the town is estimated to be 92,470. Among these 46,738 are females [23]. Currently, it has seven kebeles and that makes it the smallest administrative unit in Ethiopia. The total number of households within the seven kebeles is 24,914. Debre Markos town has one referral hospital, three public health Centers, seven private clinics and 14 health posts, seven in rural and seven in urban areas. All four public health institutions and three private clinics in the town are providing ANC services.

### Sample size and sampling techniques

All pregnant women who visited the public health institutions in the town for ANC service were included in the sample. The required sample size of eligible mothers for the study was calculated using the formula to estimate single population proportion [24]. The following assumptions were made while calculating the sample size. A 95% probability of obtaining the population proportion of pregnant mothers who experienced intimate partner violence during their pregnancy within 5% margin of error and population proportion of mothers who experienced intimate partner violence during pregnancy was assumed to be 44.5% taken from the previous study done in Oromia region, Western Ethiopia [10].

Therefore, the required sample size was 380. Expecting a 10% non-response rate, the final sample size was calculated to be 418. Accordingly, systematic random sampling technique was used to select study participants. First, all public health facilities in Debre Markos town were considered and then based on the number of pregnant women that visited the four public health facilities during the preceding month before data collection, proportional allocation of the total sample size was carried out to get the required sample size from each public health facility. The sampling interval was calculated for each health Institutions.

### Study variables

Intimate partner violence during pregnancy is an outcome variable, while others like socio-demographic, husband/partner characteristics, socio-cultural and family experience of violence and reproductive variables are explanatory variables included in the study. In this study intimate partner was considered as current spouse, co-habited (live in the same house without formal marriage), Current non-marital partners (boyfriends), former partner or spouse [21]. if the respondent says “Yes” to any one of the ranges of sexually, psychologically, and physically or any combination of the three coercive acts used against adult and adolescent women, regardless of the legal status of the relationship with current/former intimate partner, it was considered as intimate partner violence [25]. Physical violence was defined as if the study participants say **“**Yes” to one or more intentional acts of physical force or aggression such as pushing, slapping, throwing, hair pulling, punching, kicking, or burning, use of a weapon, perpetrated with the potential to cause harm, injury, disability or death towards pregnant women.

Psychological/emotional violence was defined as if the study participant says **“**Yes” to one or more acts or threats of acts, such as shouting, controlling, intimidating, humiliating, and threatening the victim. Sexual violence was defined as if the study subject says **“**Yes” to any one of the uses of force, coercion, or psychological intimidation to force the woman to engage in a sex act against her will whether or not it is completed.

### Data collection procedures

Validated instrument, based on the standard of WHO(2005) Multicounty study on women’s health and domestic violence against women[25] was used to collect data from each of the study participants. This questionnaire has four items for psychological violence, six items for physical violence, and three additional items for sexual violence. The standardized questionnaire was first prepared in English and then translated to Amharic (local language) and back to English to maintain consistency of the tool. Five female midwives for data collection and one BSC midwife for supervision were recruited from each of the public health institutions in Debre Markos town.

A face-to-face interviewer-administered questionnaire was used to collect data from all pregnant women who consented to be part of the study. Data collectors and the supervisor were trained for one day on techniques of data collection and on supervision. The principal investigator and supervisor made a day to day on-site supervision during the whole period of data collection and checked each questionnaire daily for completeness and consistency. The questionnaire was pre-tested to check the response, language clarity and appropriateness of the questionnaire while the pretest was done outside study area at Bichena with 5% of sample size on 21 women. Based on the finding from the pre-test, modification on the questionnaire was done, and arrangement of questions was revised.

### Data processing and analysis

The data were first checked manually for completeness and then coded and entered into Epi Info version 7.1.2.0. Then the data were exported to Statistical Package of Social Science (SPSS) version 20.00 for data checking, cleaning, and analysis.

Descriptive statistics (like median, interquartile range, frequencies, and percentages) were used to describe the study population in relation to dependent and independent variables. Results were presented in text, tables, graphs and charts.

Binary logistic regression (bivariable and multivariable logistic regression) was used to identify statistically significant independent variables and independent variables having a p-value less than 0.2 in the bivariable analysis were entered into multivariable logistic regression for further analysis. A p-value<0.05 in the multivariable analysis was considered as statistically significant. Hosmer-Lemeshow goodness-of-fit was used to test model fitness. Adjusted odds ratio (AOR) with 95% confidence interval was used to identify factors associated with intimate partner violence during pregnancy.

Ethical clearance was obtained from the Institutional Review Board (IRB) of University of Gondar, Institute of public health. A formal letter of study approval (letter of cooperation) was obtained from Debre Markos town health office. As per request of the investigator, Debre Markos town health office wrote a letter of permission and the letter was submitted to each public health facility focal person. After securing necessary permissions and detailed explanation of the purpose, risks, and benefits of the study to the study participants, written informed consent was obtained from all participants and only anonymous data were collected in private rooms. All data taken from the participants were kept strictly confidential and used only for the study purpose. During data collection, respondents who are victims of the violence have received appropriate counseling and care by midwives working in the ANC room.

## Results

### Socio-demographic characteristics of the study participants and their partners

A total of 418 participants were selected for this study. Of these, 409 participants were enrolled with a response rate of 97.8%. Their median age was 26 years with (IQR of 23 to 30years). Majority, 339 (82.9%) of the study participants were orthodox by religion. Nearly all 391 (95.6%) of the women were married. Regarding educational status, 128 (31.3%) of them had no formal education while 114 (27.9%) had attained secondary/ above Grade 12. Forty-six percent (46.0%) of respondents’ occupation was a housewife. Nearly all 399 (97.6%) of the respondents belonged to Amhara by ethnicity. The median monthly income of the respondents was 2500 with IQR of 1500 ETB-4000ETB. The median age of partners’ was 31years with (IQR of 28 to 37 years) and about 147 (35.9%) of partners attained more than secondary education **[Table 1].**

**Table 1:** Socio-demographic characteristics of study participants and their partners’ in Debre Markos town, Northwest, Ethiopia, April 2018(n = 409).

Characteristics	Frequency	Percentage
**Age of women in years (n = 409)**		
17–23	118	28.9
24–26	95	23.2
27–30	106	25.9
31–46	90	22.0
**Age of partners in years (n = 409)**		
20–28	110	26.9
29–31	98	24.0
32–37	107	26.1
38–60	94	23.0
**Religion(n = 409)**		
Orthodox	339	82.9
Muslim	59	14.4
Protestant	8	2.0
Catholic	3	0.7
**Residence (n = 409)**		
Rural	116	28.4
Urban	293	71.6
**Current marital status (n = 409)**		
Single	8	2.0
Married	391	95.6
Divorced	7	1.7
Widowed	1	0.2
Separated	2	0.5
**Age at marriage (in Yrs.) (n = 401)**		
<18	114	28.4
≥18	287	71.6
**Who choose her husband (n = 401)**		
Both	116	28.9
My self	88	21.9
My family	185	46.1
Partner choose	5	1.2
Partner’s family	5	1.2
My colleague	2	0.5
**Type of marriage ceremony (n = 401)**		
No marriage ceremony	29	7.2
Civil marriage	52	13.0
Religious marriage	56	14.0
Customary marriage	264	65.8
**Dowry/bride price payment (n = 401)**		
Yes	251	62.6
No	150	37.4
**Educational status of women (n = 409)**		
No formal education	128	31.3
Primary education	64	15.6
Secondary education	103	25.2
More than secondary	114	27.9
**Educational status of partner (n = 409)**		
No formal educational	123	30.1
Primary education	44	10.8
Secondary education	95	23.2
More than secondary	147	35.9
**Occupational status of women (n = 409)**		
House wife	188	46.0
Farmer	75	18.3
Student	1	0.2
Private employee	18	4.4
Government employee	79	19.3
Merchant	35	8.6
Others^a^	13	3.2
**Occupational status of partners (n = 409)**		
Farmer	107	26.2
Student	3	0.7
Private employee	93	22.7
Government employee	122	29.8
Merchant	66	16.2
Others^b^	18	4.4
**Ethnicity (n = 409)**		
Amhara	399	97.6
Tigre	5	1.2
Oromo	4	1.0
SNNP	1	0.2
**House hold average monthly income(n = 409)**		
<2500 ETB	212	51.8
≥2500ETB	197	48.2
**currently living with partner(n = 409)**		
Yes	374	91.4
No	35	8.6
**Decision maker in household (n = 374)**		
Husband	132	35.3
Wife	4	1.1
Equally	238	63.6
**Presence of mental illness (n = 409)**		
Yes	12	2.9
No	397	97.1

Others^a^_______daily laborer, unemployed, Others^b________^Driver, Deacon, daily laborer, priest

### Partner’s behavioural characteristics

**Fig 1:**
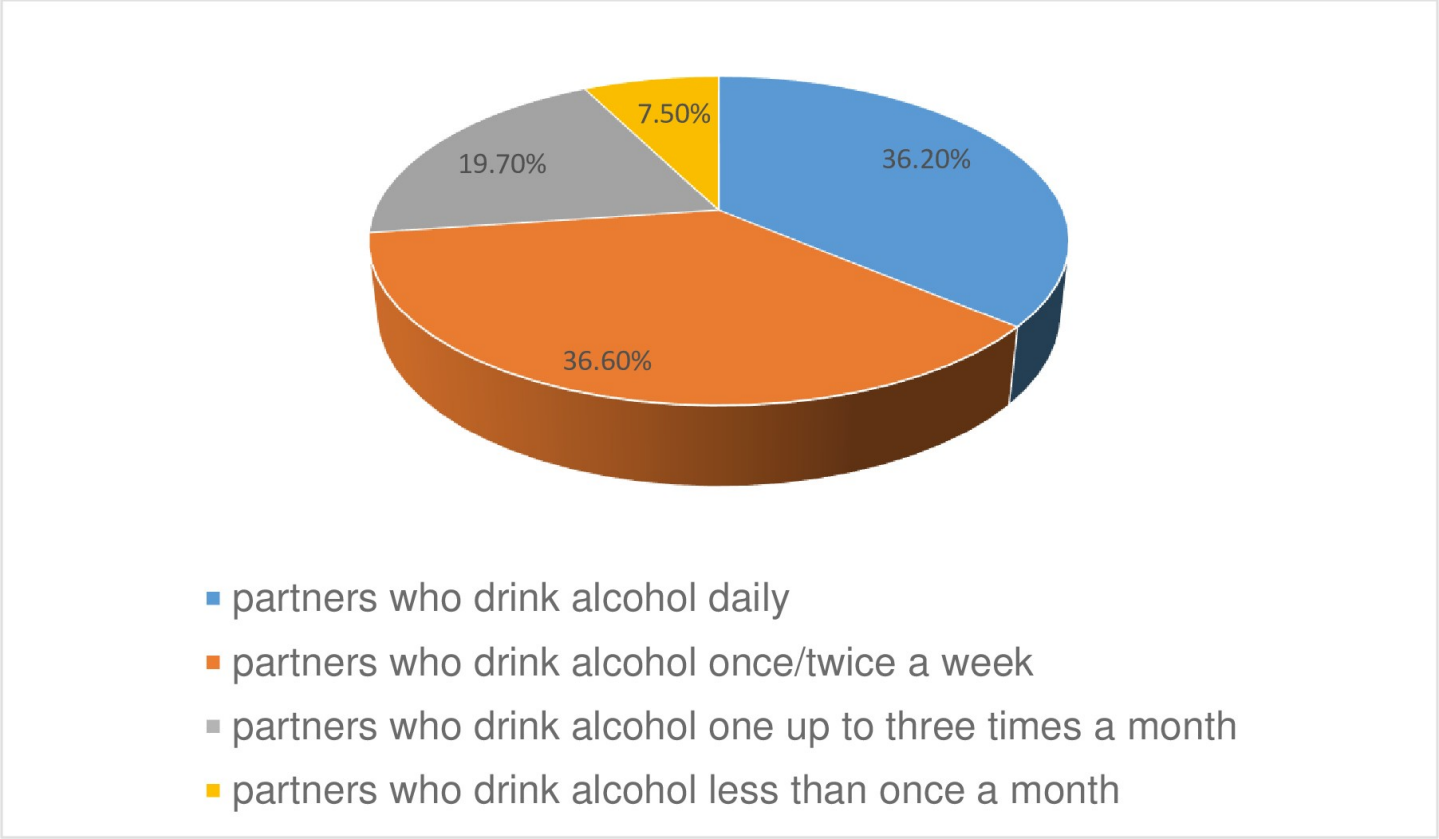
Distribution of alcohol consumption by their partners among pregnant women who came for ANC service in public health Institutions of Debre Markos town, North West, Ethiopia, April 2018.

Three hundred eighty-four (93.9%) and three hundred seventy-two (91.0%) of husbands/partners were none smokers and did not chew Kchat respectively. 310 (75.8%) of the respondents declared that their husbands have no other known girlfriends/wives. About three hundred forty-six (84.6%) participants replied that their husbands have no child from other girlfriends/wives. 254 (62.1%) of the husbands/partners were alcohol users **[Fig 1].**

### Sociocultural and family experience of violence

**Fig 2:**
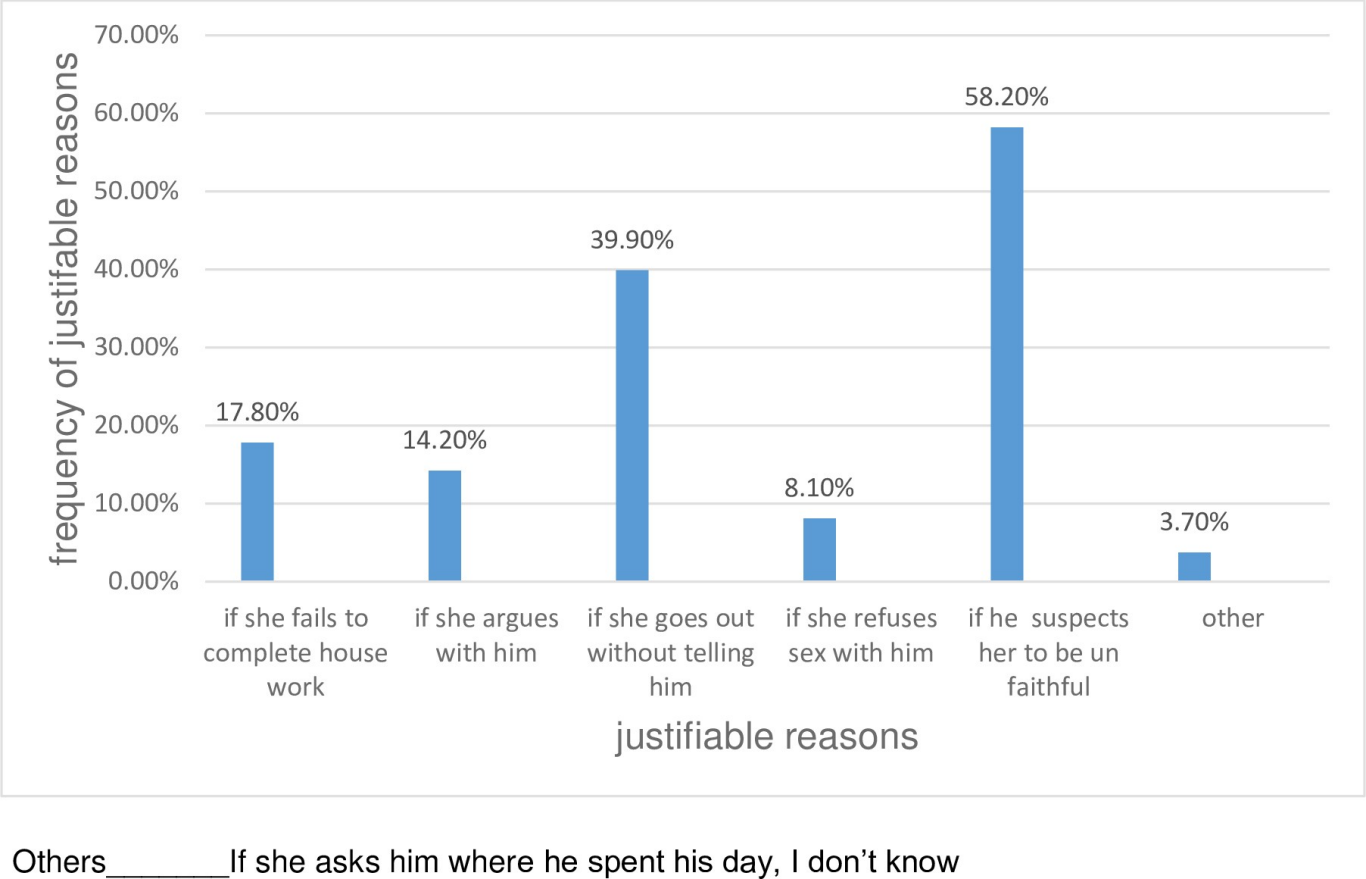
Distribution of justifiable reasons of wife-beating among pregnant women who had a positive attitude for wife beating in ANC service of public health Institutions in Debre Markos Town, North West, Ethiopia April 2018.

Parent’s history of intimate partner violence and childhood violence were assessed as predictors for intimate partner violence during pregnancy. Two hundred forty-nine (60.9%) of the study participants had no history of maternal violence by their father. However, seventy-eight (19.1%) of the respondents stated that their mothers had been beaten by their spouses. The remaining 82 (20%) of them did not witness a violence. About 371(90.7%) of the respondents responded that they had no history of violence during their childhood whereas 12(2.9%) of the study subjects reported that they faced violence during their childhood. The rest 26 (6.4%) of them did not recall a violence. The majority that is, 394(96%) of the respondents believed that the husband has the right to beat his wife during pregnancy for at least one justified reason **[Fig 2]**.

### Obstetrics related characteristics of participants

Three hundred sixty-five (89.2%) of the respondents became pregnant for the first time at the age 18 years and above and more than half of them (52.6%) had one or more pregnancies. More than half of the study participants (51.6%) started ANC follow up in the first trimester [**Table 2]**.

**Table 2:** Obstetrics characteristics of ANC attendants in Debre Markos town, Northwest, Ethiopia, April 201 (n = 409).

Variable	Frequency (n = 409)	Percentage
**Age at first pregnancy(in years)**		
<18	44	10.8
≥18	365	89.2
**Gravidity**		
Primigravida	194	47.4
Multigravida	215	52.6
**Parity**		
Nulliparous	205	50.1
Multiparous	204	49.9
**Desire of pregnancy by women**		
Yes	349	85.3
No	60	14.7
**Desire of pregnancy by partner**		
Yes	352	86.1
No	57	13.9
**First ANC initiation**		
First trimester	211	51.6
Second trimester	169	41.3
Third trimester	29	7.1
**History of abortion**		
Yes	24	5.9
No	385	94.1

### Prevalence and form of intimate partner violence during pregnancy

**Fig 3:**
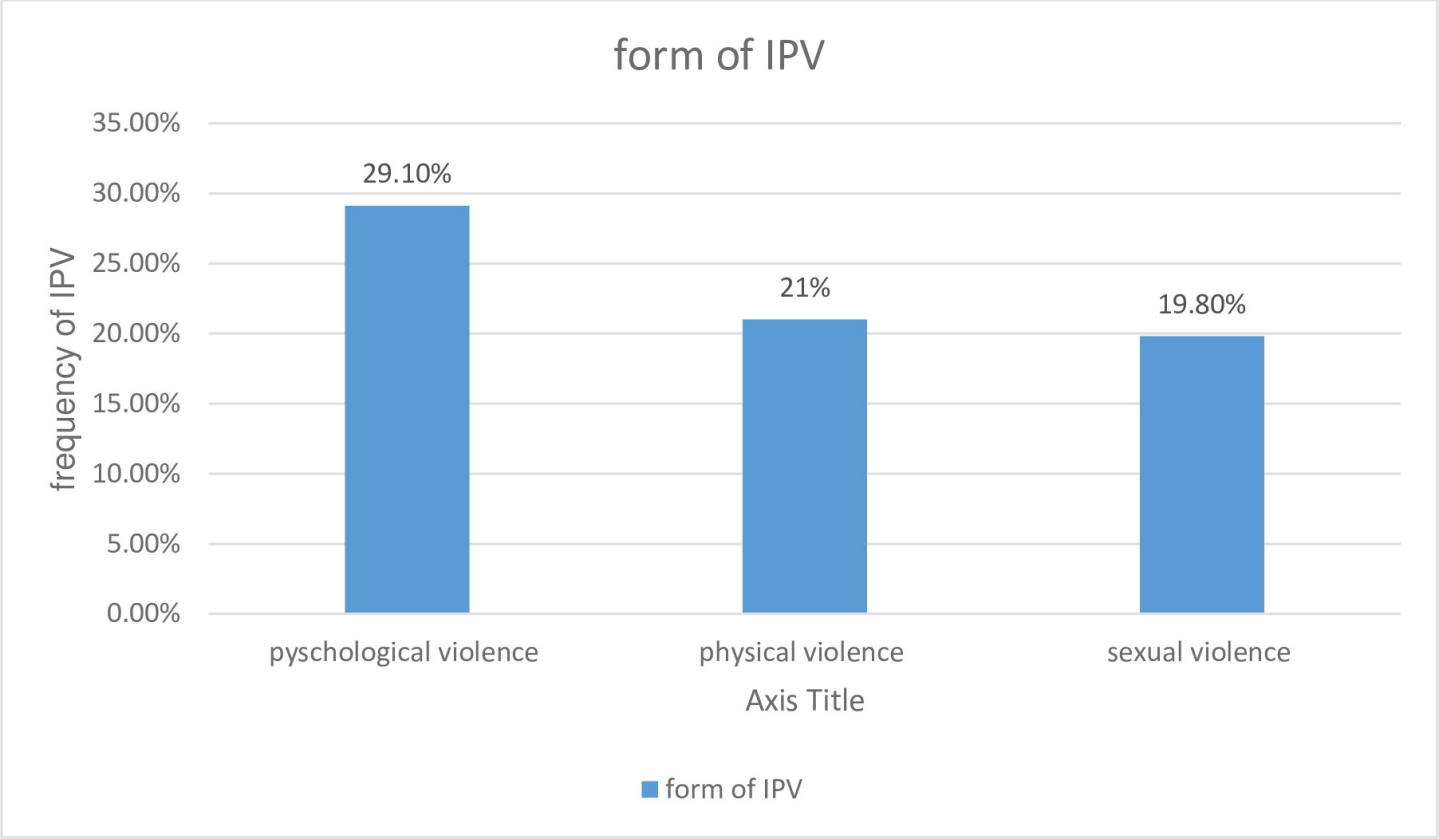
Form of intimate partner violence during pregnancy, Debre Markos town, North West, Ethiopia, April 2018.

In this study, the overall prevalence of intimate partner violence during current pregnancy was **41.1% (95% CI: 36.0–46.0) [Fig 3].**

Of this, the prevalence of psychological, physical, and sexual violence was 29.1%, 21%, 19.8% respectively. Regarding the frequencies of violence toward each item, slapping 63(15.4%) was the commonest form of violence among physical violence (forms). Having unwanted sexual intercourse because of fear of the partner 55 (13.4%) and intimidation 85 (20.8%) were commonest form of sexual and psychological/emotional violence respectively [**Table 3].**

**Table 3:** Prevalence of intimate partner violence among pregnant women in Debre Markos town, Northwest, Ethiopia, April 2018 (n = 409).

Violence item	Frequency	Percentage
**Psychological violence**	**119**	**29.1**
Insulted/made feel bad about self	62	15.2
Belittled or humiliated in front of other people	48	11.7
Scared or intimidated on purpose	85	20.8
Threatened when visiting friends/family	46	11.2
**Physical violence**	**86**	**21**
Slapped you or thrown something at you that could hurt you	63	15.4
Pushed you or shoved or pulled your hair	30	7.3
Hit you with his fist or with something else that could hurt you	21	5.1
Beaten in the abdomen	7	1.7
Choked or burnt you on purpose	13	3.2
Threatened to use or actually used a gun, knife, or any other weapon against you	19	4.6
**Sexual violence**	**81**	**19.8**
Physically forced you to have sexual intercourse	40	9.8
Having unwanted sexual intercourse because of fear from the partner	55	13.4
Forced you to do something sexual that is degrading or humiliating	53	13.0
**Overall prevalence of intimate partner violence during pregnancy**	**168**	**41.1**

### Factors associated with intimate partner violence during pregnancy

Bivariable and multivariable logistic regression analyses were done to identify factors associated with intimate partner violence during pregnancy. In bivariable analyses, age of women, residence, educational status of women, educational status of partner, occupational status of women, occupational status of partner’s, frequency of alcohol drinking by partner, who choose her husband, had other wife, had another child, gravidity, parity, first ANC initiation and desire of pregnancy by women had association with intimate partner violence during pregnancy. Only age of women, residence, who choose her partner, educational status of partner, frequency of alcohol drinking by partner and first antenatal care initiation were significantly associated with IPV in multivariable analysis. Study participants whose age was between 17–26 years were 79% less likely to have violence during pregnancy than study participants whose age was between 27–46 years (AOR = 0.21, 95%CI: 0.09–0.49) by their husbands.

Study participants who were from rural residences experienced IPV during pregnancy more than four times when compared to study participants in urban residences (AOR = 4.04, 95%CI: 1.17–13.93). Those respondents who took an active role in choosing her husband only by themselves were 3.26 times more likely to be violated by their intimate partner during pregnancy compared with those who chose equally with their husbands/partners(AOR = 3.26,95%CI:1.24–8.57).

When compared to educated, uneducated partners were 3.26 times more likely to use violence against their intimate partner during recent pregnancy (AOR = 3.26, 95%CI: 1.45–7.36). Pregnant women, whose partners drink alcohol daily, were 4.79 more likely to experience IPV during pregnancy by their husbands/partners compared with those pregnant women, whose partners drink alcohol less than three times per month. (AOR = 4.79, 95% CI: 2.08–11.04). Pregnant women, whose partners drink alcohol 1-2times/week, were 2.30 times more likely to be violated during pregnancy by their intimate partners compared with those pregnant women, whose partners drink alcohol ≤ 3 times/month (AOR = 2.30,95% CI:1.05–5.05). Pregnant women, who started their ANC during the first trimester, were 56% less likely to face IPV during their pregnancy compared to those that started their ANC during second trimester and above (AOR = 0.44, 95% CI: 0.24–0.81) **[Table 4]**.

**Table 4:** Bivariable and multivariable logistic regression analysis of factor associated with intimate partner violence among pregnant women in Debre Markos town, North West Ethiopia, 2018 (n = 409).

Variables	Intimate partner violence		COR	95%CI	AOR (95%CI)
	Yes	No			
**Age of women**					
17–26	69	144	0.47	0.31–0.70	0.21(0.09–0.49)**
27–46	99	97	1		
**Residence**					
Rural	70	46	3.03	1.94–4.72	**4.04(1.17–13.93**)*
Urban		98	195	1	
**Educational status of women**					
Uneducated	68	60	2.05	1.34–3.14	0.53(0.21–1.29)
Educated	100	181	1		
**Educational status of partner’s**					
Uneducated	73	50	2.94	1.90–4.54	3.26(1.45–7.36)*
Educated	95	191	1		
**Occupational status of women**					
House wife	74	114	1		
Farmer	46	29	2.44	1.41–4.23	0.27(0.04–1.83)
Private and Gov’t employee	31	66	0.72	0.43–1.21	0.37(0.04–3.04)
Merchant	11	24	0.71	0.33–1.53	0.32(0.04–2.46)
**Occupational status of partner’s**					
Farmer	61	46	1		
Private employee	36	57	0.48	0.27–0.84	0.57(0.09–3.70)
Gov’t employee	38	84	0.34	0.20–0.86	2.41(0.51–11.38)
Merchant	25	41	0.46	0.25–0.86	2.87(0.53–15.51)
**Who choose her husband**					
Both	37	80	1		
My self	37	57	1.40	0.80–2.48	3.26(1.24–8.57)*
My family	90	96	2.03	1.25–3.29	1.84(0.71–4.75)
**Frequency of alcohol drinking by partner**					
Daily	57	35	3.48	1.80–6.72	4.79(2.08–11.04)*
1–2 times/week	42	51	1.76	0.92–3.37	2.30(1.05–5.05)*
≤3 times/month	22	47	1		
**Had other wife**					
Yes	47	52	1.41	0.90–2.23	1.14(0.50–2.56)
No	121	189	1		
**Had other child**					
Yes	36	27	2.16	1.26–3.73	1.74(0.68–4.44)
No	132	214	1		
**Gravidity**					
Primi gravida	73	121	0.76	0.51–1.13	2.66(0.52–13.62)
Multi gravida	95	120	1		
**Parity**					
Nulliparous	77	128	0.75	0.50–1.10	0.88(0.18–4.35)
Multiparous	91	113	1		
**First ANC initiation**					
First trimester	66	145	0.43	0.29–0.64	0.44(0.24–0.81)*
Second trimester and above	102	96	1		
**Desire of pregnancy by women**					
No	30	30	1.53	0.29–0.64	1.03(0.44–2.43)
Yes	138	211	1		

ANC = Antenatal Care, COR = Crude odds ratio, AOR = Adjusted odds ratio, CI = Confidence interval, 1 = reference category.

** P ≤0.001

*p<0.05

## Discussion

Intimate partner violence during pregnancy is the serious form of violence that negatively affects the health of women and the fetus she bears.

The current study was conducted to determine the prevalence of intimate partner violence among pregnant women who were attending antenatal care service in public health institutions of Debre Markos Town. The study also focused on predictors that influence the occurrence of intimate partner violence during pregnancy.

This study demonstrated that the overall prevalence of intimate partner violence during recent pregnancy was found to be 41.1% (95% CI: 36.0–46.0) and it was associated with the age of women between 27-46yers, rural residency, uneducated partner, choice of partner by the women, frequent alcohol drinking by partner, late initiation of antenatal care,. Out of the overall prevalence of 41.1%, 21% was physical violence, 19.8% was sexual violence, and 29.1% was psychological violence. This finding (41.1%) is comparable with studies carried out in Abay Chomen district, Western Ethiopia (44.5%) [10], Kenya(37%) [22], Abakaliki, Southeast, Nigeria (44.6%) [26], but higher compared to studies done in china (7.7%) [27],Namibia (8%) [28],Tanzania (27%)[17], South Africa (20%)[29] and Hossana, Ethiopia (23%) [30].

The possible explanation for the observed variation from a study in Hossana might be due to an intercultural difference between the two settings. Besides, the probable cause for the discrepancy may be owing to the difference in the sample size they used.

The high prevalence rate in the current study may be because of the presence of traditional gender norms that support wife beating, and the women themselves accept wife beating in this catchment area. This is also supported by the available evidence that noted the widespread relationship between wife beating and high prevalence of intimate partner violence [31]. Moreover, in Ethiopia, intimate partner violence has been considered as a culturally specific phenomenon and is influenced by religion and the sociocultural context [32].

In contrast, the prevalence of intimate partner violence during pregnancy in this study is lower than studies conducted in Gambia (61.8%) [33], Oyo East local government, Nigeria(72%) [34],Zimbabwe(65.4%) [14]. The gap between our study and a study in Oyo East Local Government, Nigeria may be because of difference in violence measure as the study in Nigeria used additional items of psychological violence than our study to measure IPV. The study in Oyo has treated emotional and psychological violence separately and this would have resulted in high prevalence of IPV. The possible difference observed from the study in Zimbabwe might be due to the timing of interviewing as the study in Zimbabwe was conducted during the post-natal period, which may provide an opportunity to detect the violence during the full course of pregnancy [26]. Overall, the possible explanation for the variation may be due to the difference in the definition of IPV used to measure violence as there are lack of standardized definitions and lack of tools to diagnose violence, cultural variation among countries, difference in the source population, the study design, the availability of information on sexual and reproductive health issues and accessibility of information on gender-based issues. The prevalence of psychological violence in the current study (29.1) is higher than studies conducted in Abay Chomen, Oromia region (16%) [10], Hossana (20%) [30] but consistent with Kenyan study (29%) [35]. The prevalence of physical violence in our study (21%) is lower than the study in Abay Chomen (29%) [10] but higher than studies carried out in Yirgalem town (12%)[36], Shirie Endesillasie (20.6%)[21] and Kenya (10%)[35]. The prevalence of sexual violence in our study showed a high prevalence compared to the study in Hossana (12%)[30] but lower than the study done in Abay Chomen (30%) [10].

Regarding factors associated with IPV during pregnancy, study participants who were from rural residences experienced IPV during pregnancy more than four times higher compared to urban residences (AOR = 4.04, 95%CI: 1.17–13.93). Similar findings were reported from previous studies done in Shirie, Ethiopia [21] and Bangladesh [37]. This could be due to the fact that women who are from rural residencies might not have access to a range of information that deal with women right of equality with their intimate partner, violence reduction mechanisms and may be more influenced by traditional influences.

Our finding showed that pregnant women whose partners usually drink alcohol (2–3 times per week) were 2.30 times (AOR = 2.30, 95% CI: 1.05–5.05) more likely to experience IPV during pregnancy by their husbands/partners compared with those pregnant women whose partners rarely drink alcohol (less than or equal to three times per month). This risk increased to more than two times (AOR = 4.79, 95% CI: 2.08–11.04) for those pregnant women having partners who frequently (daily) drink alcohol. This result is consistent with other studies done in Kenya[22], Zimbabwe [38], Rwanda [20], Ethiopia [39]. This may be due to the fact that excessive alcohol drinking can cause aggression, altered mental judgement, and this increases the likelihood of violence. Furthermore, some persons may intentionally use alcohol in order to hide behind the alcohol so as to engage in anti-social behaviors like violence against their partners [20].

In this study, partner’s educational status was found to be significantly associated with IPV during pregnancy noting that uneducated partners were 3.26 times more likely to use violence against their intimate partners during recent pregnancy (AOR = 3.26, 95%CI: 1.45–7.36). This is in line with studies conducted in Kenya [22], Bangladesh [37], Nigeria [26], Hossana [30], Yirgalem [36] which revealed that a partner who attended tertiary education is protective against intimate partner violence during pregnancy. This could be due to the fact that uneducated partners are more likely to have ingrained traditional perceptions concerning gender equality. This is supported by a study reported that most of the intimate partners who beat their wives are uneducated and account traditional belief of wife beating as a norm [21]. On the contrary to this study, a study done in Nigeria showed that intimate partners who had no formal education contributed little to the prevalence of IPV[40].

This study also found a significant association between age of women and violence during pregnancy by their intimate partners. Study participants whose age was between 17–26 years were 79% less likely to have faced violence during pregnancy by their husband than study participants whose age was between 27–46 years (AOR = 0.21, 95%CI: 0.09–0.49). This finding is supported by Ethiopia Demographic and health survey (EDHS) 2016. The possible explanation might be as the age of women increases family size also increases which may result in economic crisis and finally end up with spousal disagreement. In our study, the timing of ANC initiation for the experience of IPV during pregnancy is found to be significantly associated. Pregnant women who started their ANC during the first trimester were 56% less likely to face IPV during their pregnancy compared to those that started their ANC during second and third trimester (AOR = 0.44, 95% CI: 0.24–0.81). This finding is comparable with other studies carried out in South Africa [41] and Gondar [42]. This may be due to the fact that early initiation of ANC increases the number of visits which in turn increase the women’s chance of getting information from health care providers about sexual and reproductive health including violence. An interesting finding in this study was the influence of women’s participation in her choice of husband on the experience of intimate partner violence during pregnancy. Those respondents who took an active role in choosing her partner only by themselves were 3.26 times more likely to be violated by their intimate partners during pregnancy compared with those who chose equally with husbands/partners(AOR = 3.26,95%CI:1.24–8.57). There is no study consistent with this finding as per our review. This could be due to the fact that if both spouses do not love each other, that is, if the husband loves his wife less than the women loves him at the time of marriage, the women may want to be pregnant from their partner in order not to lose him; however, the partner may not demand the pregnancy and become more likely to abuse his wife during pregnancy.

However, occupational status and household average monthly income were found to have no significant association with intimate partner violence in our study. Studies that are in line with our finding revealed that occupation and socioeconomic status had minimal effect or do not reduce the likelihood of intimate partner violence during pregnancy [43] especially for women in low-income countries (pregnant women work largely in informal sectors with low paid jobs) and in patriarchal societies (pregnant women are usually exposed to the same patriarchal social structures at the workplace that may further strengthen the myth of male superiority). Gravidity, parity, desire of pregnancy, educational status of the respondent, the age of partner, had an extra wife and children are not also significantly associated with the experience of intimate partner violence during pregnancy in this study.

### Strength and weakness of the study

The main strength of this study is that, the use of validated instruments of WHO multi-country study on violence against women. This study also has some important limitations that should be considered when interpreting the results. Since the topic is sensitive some respondents may not be volunteer to disclose their violence (social desirability bias), which in turn leads to underreporting.

## Conclusions

The result of this study identified that intimate partner violence during current pregnancy was found to be high. Since more than one out of four women were abused by their intimate partners. Of those women with a history of IPV psychological violence was the commonest form followed by physical and sexual violence.

Lower educational status of partners, rural residency, frequent alcohol drinking by partner, late initiation of antenatal care, the age of women, who choose her husband/partner were important predictors of intimate partner violence during current pregnancy. Awareness creation to the community has to be made about the negative health outcomes of intimate partner violence during pregnancy and interventions that would address the specified risk factors are recommended.

## Supporting information

S1 Table English version questionnaire. (DOCX)

S2 Table Amharic version questionnaire. (DOCX)

## Abbreviations

ANC: Antenatal Care
AOR: Adjusted Odds Ratio
CI: Confidence Interval
DV: Domestic Violence
EDHS: Ethiopian Demographic Health Survey
EPI: Epidemiological Information
ETB: Ethiopian Birr
GTB: Growth and Transformation Plan
IPV: Intimate Partner Violence
OR: Odds Ratio
SPSS: Statistical Package for Social Science
WHO: World Health Organization
