# Supplementary material for: Intimate partner violence and associated factors among pregnant women attending antenatal care service in Debre Markos town health facilities, Northwest Ethiopia
Source: PLoS One. 2019 Jul 1;14(7):e0218722. doi: 10.1371/journal.pone.0218722 (PMC6602189; doi:10.1371/journal.pone.0218722)
Supplement: S1 Table — (DOCX) [file pone.0218722.s001.docx]

**Annex II:** **English Version Questionnaire**

**Part I**: **Socio- demographic characteristics** **of pregnant women and her current/most recent partner in public health institutions of Debre Markos town, North West Ethiopia, 2018**

| **S/no** | **Questions** | **Answers/choices** | | | **Skip** |
| --- | --- | --- | --- | --- | --- |
| 101 | Current age? | ___________ year | | |  |
| 102 | Partner’s age? | ___________year | | |  |
| 103 | What is your religion? | 1. Orthodox 2. Muslim 3. Protestant 4. Catholic 5. Others (Specify)________ | | |  |
| 104 | Residence? | 1. Rural 2. Urban | | |  |
| 105 | Current Marital status? | 1. Single 2. Married 3. Divorced 4. Widowed 5. Separated | | | If Q no 105 answer is not 2 skipping until Q no 109 |
| 106 | If you are married, age at marriage? | ____________ Year | | |  |
| 107 | Who choose your current/most recent husband? | 1. Both 2. My self 3. My family 4. Partner choose 5. Partner’s family 6. Other (specify)__________ | | |  |
| 108 | What type of marriage ceremony did you have to formalize the union? | 1. None 2. Civil marriage 3. Religious marriage 4. Customary marriage 5. Others (specify)________ | | |  |
| 109 | Did your marriage involve dowry/bride price payment? | 1. Yes 2. No 3. I don’t know | | |  |
| 110 | What is your educational status? | 1. No formal education 2. Primary education (Grade1_6) 3. Secondary education(7-12) 4. More than secondary(above grade 12) | | |  |
| 111 | What is your partner’s educational status? | 1. No formal education 2. Primary education(Grade1_6) 3. Secondary education(7-12) 4. More than secondary(above Grade 12) | | |  |
| 112 | What is your occupation? | 1. House wife 2. Farmer 3. Student 4. Private employee 5. Government employee 6. Merchant 7. Others (specify)________ | | |  |
| 113 | What is your partner’s occupation? | 1. Farmer 2. Student 3. Private employee 4. Government employee 5. Merchant 6. Others (specify)__________ | | |  |
| 114 | Ethnicity | 1. Amhara 2. Tigre 3. Oromo 4. Others (specify)__________ | | |  |
| 115 | How much is your family average monthly income? | ____________in ETB | | |  |
| 116 | With whom you are currently living? | 1. My partner 2. My parents 3. Any of my relatives 4. My partner’s family 5. Others (specify)_______ | | | If not 1 skip to Q118 |
| 117 | If your answer in Q no 116 is 1 who is the decision maker in all affairs of the household? | 1. Husband 2. Wife 3. Equally | | |  |
| 118 | Have you diagnosed mental illness by health care provider currently or in the past life? | 1. Yes 2. No | | |  |
| **Part II ፡Current husband /partner’s behaviour question** | | | | | |
| 201 | Does/did your husband/partner drink alcoholic beverages like Areke, Tella, and Tej? | 1. Yes 2. No 3. I don’t know | | | If 2/3 skip to Q 203 |
| 202 | How frequently do your partner drink alcoholic beverages like Areke, Tella, and  Tej? | 1. Daily 2. 1-2 times/week 3. 1-3 times/month 4. Less than 1 times in a month 5. Others (specify)________ | | |  |
| 203 | Does/did your husband/partner chew Kchat/Qat? | 1. Yes 2. No 3. I don’t know | | | If 2/3 skip to Q 205 |
| 204 | How frequently do your partner chew Kchat/Qat? | 1. Daily 2. 1-2 times/week 3. 1-3 times/month 4. Less than 1 times in a month 5. Others (specify)_______ | | |  |
| 205 | Does/did your husband/partner smoke cigarette? | 1. Yes 2. No 3. I don’t know | | | If 2/3 skip to Q 207 |
| 206 | How frequently do your husband smoke cigarette? | 1. Daily 2. 1-2 times /week 3. 1-3 times/month 4. Less than one 1 times in month 5. Others (specify)________ | | |  |
| 207 | Has your current/most recent husband/partner had a relationship with any other women while being with you/extra marital sex? | 1. Yes 2. No 3. I don’t know | | | If the answer is no skip to Q 209 |
| 208 | How many wives does/did he have (including yourself)?polygamy marriage | 1. Number of wives_______ 2. I don’t know | | |  |
| 209 | Has your current/most recent husband/partner had children with any other women while being with you? | 1. Yes 2. No 3. I don’t know | | |  |
| **Part III :socio-cultural and family experience of violence** | | | | | |
| 301 | When you were a child, was your mother hit by your father (her husband)? | 1. Yes 2. No 3. Not remember | | |  |
| 302 | As far as you know, did your father ever beat your mother? | 1. Yes 2. No 3. Not remember | | |  |
| 303 | Did you experience any violence during child hood? | 1. Yes 2. No 3. Not remember | | |  |
| 304 | According to your opinion, what are a good reasons for a husband to beat his wife?  (More than one answer is possible) | 1. Fails to complete house work 2. Argues with him 3. Goes out without telling him 4. Refuses sex with him 5. He suspects /founds her to be un faithful 6. Others (specify)_________ | | |  |
| **Part IV: Obstetrics/reproductive related questions** | | | | | |
| 401 | Age at first pregnancy? | _____________Year. | | |  |
| 402 | How many pregnancies do you have (number of total pregnancies)? Gravidity? | ___________pregnancy/pregnancies | | |  |
| 403 | How many children’s do you have? Parity? | ______________child/children | | |  |
| 404 | Did you desire this pregnancy? | 1. Yes 2. No | | |  |
| 405 | Did your husband want/ desire this pregnancy? | 1. Yes 2. No 3. I don’t know | | |  |
| 406 | When did/do you start ANC follow up? | ___________ Month | | |  |
| 407 | Have you ever aborted? | 1. Yes 2. No | | |  |
| **Part V**፡ **about psychological(emotional ),physical and sexual violence:** | | | | | |
| **Psychological(emotional) violence** | | | | | |
| Has your partner ever…… | | | | | |
| 501 | Did your partner/ husband ever perform any violence in your life time? | | | 1. Yes 2. No |  |
| 502 | Did your partner/ husband ever perform any violence when you were pregnant? | | | 1. Yes 2. No |  |
|  |  | Has this happened during the current pregnancy  **Yes**  **No** | | Has this happened during the previous pregnancies  (if primi gravid skip)  **Yes**  **No** |  |
| 503 | Insulted/made feel bad about your self | 1 2 | | 1 2 |  |
| 504 | Belittled or humiliated in front of other people |  | |  |  |
| 505 | Scared or intimidated on purpose | 1 2 | | 1 2 |  |
| 506 | Threatened when asking friends/family | 1 2 | | 1 2 |  |
| **Physical violence** | | | | | |
| Has your partner ever…… | | | | | |
| 507 | Slapped you or thrown something at you that could hurt you? | 1 2 | 1 2 | |  |
| 508 | Pushed you or shoved or pulled your hair? | 1 2 | 1 2 | |  |
| 509 | Hit you with his fist or with something else that could hurt you? | 1 2 | 1 2 | |  |
| 510 | During pregnancy, hit  your abdomen with a fist  Or with something else? | 1 2 | 1 2 | |  |
| 511 | Choked or burnt you on purpose? | 1 2 | 1 2 | |  |
| 512 | Threatened to use or actually used a gun, knife, or any other weapon against you? | 1 2 | 1 2 | |  |
| **Sexual violence** | | | | | |
| Has your partner ever…… | | | | | |
| 513 | Physically forced you to have sexual intercourse | 1 2 | 1 2 | |  |
| 514 | Having unwanted sexual intercourse because of fear from the partner | 1 2 | 1 2 | |  |
| 515 | Forced you to do something sexual that is degrading or humiliating | 1 2 | 1 2 | |  |

THE END

Thank you !!!
