# Supplementary material for: Intimate partner violence and associated factors among pregnant women attending antenatal care service in Debre Markos town health facilities, Northwest Ethiopia
Source: PLoS One. 2019 Jul 1;14(7):e0218722. doi: 10.1371/journal.pone.0218722 (PMC6602189; doi:10.1371/journal.pone.0218722)
Supplement: S2 Table — (DOCX) [file pone.0218722.s002.docx]

Annex IV: **የአማርኛ መጠይቅ**

**ክፍል 1፡ ማህበራዊ ነክ መረጃዎች**

| **ተ.ቁ** | **ጥያቄዎች** | **ምላሽ** | **ይለፉት** | |
| --- | --- | --- | --- | --- |
| 101 | አሁን እድሜዎ ስንት ነዉ? | ________አመት |  | |
| 102 | አሁን የትዳር ጓደኛዎ እድሜ ስንት ነዉ? | ________አመት |  | |
| 103 | ሐይማኖትዎ ምንድን ነዉ? | 1. ኦርቶዶክስ 2. ሙስሊም 3. ፕሮቴስታንት 4. ካቶሊክ 5. ሌላ ካለ ይግለፁ____________ |  | |
| 104 | መኖርያ ቤት? | 1. ገጠር 2. ከተማ |  | |
| 105 | አሁን ያልዎት የጋብቻ ሁኔታ? | 1. ያላገባች 2. ያገባች 3. የፈታች 4. የሞተባት 5. ተለያይቻለሁ | መልስዎ 2 ካልሆነ ወደ ቁጥር 109 ይሂዱ | |
| 106 | ተራ ቁጥር 105 መልስዎ አግብቻለሁ ከሆነ ሲያገቡ የነበርዎት እድሜ ስንት ነበር? | __________አመት |  | |
| 107 | የአሁኑ/የቅርብ የትዳር ጓደኛዎን የመረጠልዎት ማን ነዉ ? | 1. ሁለታችንም 2. እኔ 3. ቤተሰቦቼ 4. የትዳር ጓደኛየ 5. የትዳር ጓደኛየ ቤተሰቦች 6. ሌላ ካለ ይግለፁ----- |  | |
| 108 | ጋብቻዉን ህጋዊ ለማድረግ ምን አይነት የጋብቻ ስነ-ስርዓት ፈፀማችሁ? | 1. ምንም 2. የፍትሐ ብሔር ጋብቻ 3. ሃይማኖታዊ ጋብቻ 4. ባህላዊ ጋብቻ 5. ሌላ ካለ ይግለፁ----- |  | |
| 109 | በሰርጋችሁ ወቅት ጥሎሽ ተጥሎልሽ ነበርን ? | 1. አዎ 2. የለም 3. አላዉቅም |  | |
| 110 | የትምሕርት ደረጃዎት? | 1. መደበኛ ትምህርት ያልተማሩ 2. አንደኛ ደረጃ(1-6) 3. ሁለተኛ ደረጃ(7-12) 4. ከ2ኛ ደረጃ በላይ(ከ12ኛ በላይ) |  | |
| 111 | የትዳር ጓደኛዎ የትምሕርት ደረጃ? | 1. መደበኛ ትምህርት ያልተማረ 2. አንደኛ ደረጃ(1-6) 3. ሁለተኛ ደረጃ(7-12) 4. ከ2ኛ ደረጃ በላይ(ከ12ኛ በላይ) |  | |
| 112 | የስራ ሁኔታዎት ምንድን ነዉ? | 1. የቤት እመቤት 2. ገበሬ 3. ተማሪ 4. የግል ተቀጣሪ 5. የመንግስት ተቀጣሪ 6. ነጋዴ 7. ሌላ ካለ ይግለፁ----- |  | |
| 113 | የትዳር ጓደኛዎ የስራ ሁኔታ ? | 1. ገበሬ 2. ተማሪ 3. የግል ተቀጣሪ 4. የመንግስት ተቀጣሪ 5. ነጋዴ 6. ሌላ ካለ ይግለፁ----- |  | |
| 114 | ብሄርዎ ምንድን ነዉ ? | 1. አማራ 2. ትግሬ 3. ኦሮሞ 4. ሌላ ካለ ይግለፁ----- |  | |
| 115 | የቤተሰብዎት አማካይ የወር ገቢ ስንት ነዉ? | _____________________ በብር |  | |
| 116 | አሁን የሚኖሩት ከማን ጋር ነዉ? | 1. ከትዳር ጓደኛየ ጋር 2. ከቤተሰቦቼ ጋር 3. ከዘመዶቼ ጋር 4. ከትዳር ጓደኛየ ቤተሰቦች ጋር 5. ሌላ ካለ ይግለፁ----- | መልስዎ 1 ካልሆነ ወደ ተ.ቁ 118 ይሂዱ | |
| 117 | በተ.ቁ 116 መልስዎ 1 ከሆነ በቤት ዉስጥ ባሉጉዳዮች ላይ ዉሳኔ ሰጪ/ኃላፊ ማን ነዉ ? | 1. ባለቤቴ 2. እኔ 3. ሁለታችንም |  | |
| 118 | በአሁኑ ወቅት ወይም በህይዎትዎ በጤና ባለሙያ የተረጋገጠ የአዕምሮ ህመም አለብዎት ተብለዉ ያዉቃሉ? | 1. አዎ 2. አላዉቅም |  | |
| **ክፍል ሁለት፡ የአሁኑ የትዳር ጓደኛ/የትዳር አጋር ባህሪያትን የሚያሳይ መረጃ** | | | | |
| 201 | የትዳር ጓደኛዎ አልኮል መጠጥ ጠጥተዉ ያዉቃሉ/አረቄ፣ጠላ፣ጠጅ? | 1. አዎ 2. አይጠጣም 3. አላዉቅም | መልስዎ 2/3 ከሆነ ወደ ተ .ቁ 203 ይሂዱ | |
| 202 | ተራ ቁጥር 201 መልስዎ አዎ ከሆነ በሳምንት/ በወር ስንት ጊዜ? | 1. በየቀኑ 2. ከ 1-2 ጊዜ በሳምንት 3. ከ 1-3 ጊዜ በወር 4. በወር ከ 1 ጊዜ በታች 5. ሌላ ካለ ይግለፁ----- |  | |
| 203 | የትዳር ጓደኛዎ አደንዛዥ ዕፅ ወይም ጫት ቅመዉ ወይም ተጠቅመዉ ያዉቃሉ ? | 1. አዎ 2. አይቅምም 3. አላዉቅም | መልስዎ 2/3 ከሆነ ወደ ተ .ቁ 205 ይሂዱ | |
| 204 | ተራ ቁጥር 203 መልስዎ አዎ ከሆነ በሳምንት/በወር ስንት ጊዜ? | 1. በየቀኑ 2. ከ 1-2 ጊዜ በሳምንት 3. ከ 1-3 ጊዜ በወር 4. በወር ከ 1 ጊዜ በታች 5. ሌላ ካለ ይግለፁ----- |  | |
| 205 | የትዳር ጓደኛዎ ሲጋራ አጪሰዉ ያዉቃሉ? | 1. አዎ 2. አያጨስም 3. አላዉቅም | መልስዎ 2/3 ከሆነ ወደ ተ .ቁ 207 ይሂዱ | |
| 206 | ተራ ቁጥር 205 መልስዎ አዎ ከሆነ በሳምንት/በወር ስንት ጊዜ? | 1. በየቀኑ 2. ከ 1-2 ጊዜ በሳምንት 3. ከ 1-3 ጊዜ በወር 4. በወር ከ 1 ጊዜ በታች 5. ሌላ ካለ ይግለፁ----- |  | |
| 207 | የትዳር ጓደኛዎ ከእርስዎ ጋብቻ ላይ ሌላ ሚስት/የትዳር ጓደኛ አላቸዉ/ነበረዉ? | 1. አዎ 2. የለዉም 3. አላዉቅም | መልስዎ 2/3 ከሆነ ወደ 209 | |
| 208 | ተራ ቁጥር 207 መልስዎ አዎ ከሆነ ምን ያህል ሚስቶች አሉት ? (እራስዎን ጨምሮ) | 1. ________________ 2. አላዉቅም |  | |
| 209 | የትዳር ጓደኛዎ ከእርስዎ ዉጪ ከሌላ ሴት ልጅ አላቸዉ/ነበራዉ? | 1. አዎ 2. የለዉም 3. አላዉቅም |  | |
| **ክፍል ሶስት᎓ ማህበራዊ-ባህላዊ**  **እና በቤተሰብ ውስጥ የሚደረግ ጾታዊ ጥቃት ልምድ ጋር የተያያዙ ጥያቄዎች** | | | | |
| 301 | በልጅነትሽ ጊዜ እናትሽን አባትሽ ይደበድባት ነበርን ? | 1. አዎ 2. አይደበድባትም ነበር 3. አላስታዉስም |  | |
| 302 | እስከሚያውቁት ድረስ አባትሽ እናትሽን ደብድቧት ያዉቃል? | 1. አዎ 2. አያዉቅም 3. አላስታዉስም |  | |
| 303 | በልጅነትዎ ጊዜ ፆታዊ ጥቃት አጋጥምዎት ያዉቅ ነበር ? | 1. አዎ 2. አያዉቅም 3. አላስታዉስም |  | |
| 304 | እንደ እርስዎ ሀሳብ ባል ሚስቱን ቢመታት ትክክል ነዉ የሚሉት መቸ ነዉ? | 1. ቤት ዉስጥ መስራት ያለባትን ካልሰራች 2. ከእሱ ጋር ከተከራከረች 3. ሳትነግረዉ ከሄደች 4. የግብረ ስጋ ግንኙነት ለመፈጸም ፈቃደኛ ካልሆነች 5. ታማኝ ካልሆነች/አይደለችም ብሎ ከተጠራጠራት 6. ሌላ ካለ ይግለፁ----- | |  |
| **ክፍል IV፡ከወሊድ ጋር የተያያዙ ጥያቄዎች** | | | | |
| 401 | የመጀመሪዎን ሲያረግዙ የነበርዎት እድሜ ስንት ነበር? | __________አመት |  | |
| 402 | ይህ ስንተኛ አርግዝናዎ ነዉ | _______ኛ እርግዝና |  | |
| 403 | ስንት ልጆች አለዎት (**በህይዎት ያሉ)** ? | _____________ልጆች |  | |
| 404 | ይህንን እርግዝና አቅደዉት/ፈልገዉት ነዉያረገዙ? | 1. አዎ 2. አይደለም |  | |
| 405 | ይህንን እርግዝና የትዳር ጓደኛዎ ፈልጎት /አቅዶትነዉ ያረገዙ? | 1. አዎ 2. አይደለም 3. አላዉቅም |  | |
| 406 | የቅድመ ወሊድ ክትትል የጀመሩ መቸ ነዉ? | ________ወር |  | |
| 407 | ዉርጃ/ የፅንስ ማቋረጥ አጋጥምዎት ያዉቃል? | 1. አዎ 2. አላዉቅም |  | |
| **ክፍል 5 ፡መልስ ሰጪዋ ስለ ሥነ ልቦናዊ (ስሜታዊ), አካላዊ እና ጾታዊ ጥቃትን በተመለከተ** | | | | |
| 501 | በህይዎት ዘመንዎ የትዳር ጓደኛዎ ፆታዊ ጥቃት ፈፅሞብዎት ያዉቃል ? | 1. አዎ 2. አያዉቅም |  | |
| 502 | በእርግዝናዎ ጊዜ የትዳር ጓደኛዎ ፆታዊ ጥቃት ፈፅሞብዎት ያዉቃል ? | 1. አዎ 2. አያዉቅም |  | |
|  |  | ይህ የሆነዉ በአሁኑ አርግዝና ነዉ።  **አዎ**  **አይደለም** | ይህ የሆነዉ በባለፈዉ አርግዝናነዉ(**የመጀመሪያዎ ከሆነ ይለፉት**)።  **አዎ**  **አይደለም** | |
| **የስነ- ልቦና (ስሜታዊ) ጥቃትን በተመለከተ** | | | | |
| 503 | አንቋሽሾት ወይም ስለ ራስዎ  መጥፎ ስሜት እንዲሰማዎት አድርጎት ያዉቃል? | 1 2 | 1 2 | |
| 504 | ከሌሎች ሰዎች ፊት ሰድቦት፡  አሳፍርዎት ወይም ክብርዎትን ለመንካት ሞክሮ ያዉቃል? | 1 2 | 1 2 | |
| 505 | ሆን ብሎ አስፈራርትዎት ያዉቃል ለምሳሌ በአይን አስተያየቱ ወይም በአነጋገሩ? | 1 2 | 1 2 | |
| 506 | ጓደኞችዎን ወይም ቤተሰቦትን አንዳይጠይቁ ከልክዎት ያዉቃል? | 1 2 | 1 2 | |
| **አካላዊ ጥቃትን በተመለከተ** | | | | |
| 507 | በጥፊ መትትዎት ወይም የሚጎዳ ነገር ወርዉሮቦት ያዉቃል? | 1 2 | 1 2 | |
| 508 | ገፍቶዎት፤ገፍትሮዎት ወይም ጸጉርዎን ጎትቶዎት ያዉቃል? | 1 2 | 1 2 | |
| 509 | በቦክስ ፣ በቡጢ ወይም በሌላ ነገር መትቶዎት ያዉቃል? | 1 2 | 1 2 | |
| 510 | በእርግዝናዎ ጊዜ ሆዶ ላይ ረግጦዎት ወይም መትቶዎት ያዉቃል? |  |  | |
| 511 | አንገትዎትን አንቆ፣አፍኖ ወይም ሆን ብሎ በአሳት አቃጥልዎት ያዉቃል? | 1 2 | 1 2 | |
| 512 | በስለት/በጩቤ ፡በሽጉጥ ፡  በጠመንጃ᎓በቢላዋ ወይም ሌላ የጦር መሣሪያ አስፈራርቶት ወይም ጎድትዎት ያዉቃል? | 1 2 | 1 2 | |
| **ወሲባዊ ጥቃትን በተመለከተ** | | | | |
| 513 | አርስዎ ሳይፈልጉ ጉልበት በመጠቀም የግብረ-ስጋ ግንኙነት እንዲፈጽሙ አድርጎት ያዉቃል? | 1 2 | 1 2 | |
| 514 | አንድ ነገር ያደርገኛል ብለዉ በመፍራት ፍላጎት ሳይኖሮት የግብረ-ስጋ ግንኙነት አድርገዉ ያዉቃሉ ? | 1 2 | 1 2 | |
| 515 | ከፍላጎቶ ዉጪ ሰዉነት በመነካካት ወይም የማሻሸት ሁኔታ አጋጥምዎት ያዉቃል ? | 1 2 | 1 2 | |

**ተፈጸመ**

**ስለመልካም ትብብርዎ ከልብ አመሰግናለሁ።**
